# Mechanism of action of lncRNA-NEAT1 in immune diseases

**DOI:** 10.3389/fgene.2025.1501115

**Published:** 2025-03-05

**Authors:** Ruo-Xuan Zhang, Zi-Xuan Zhang, Xiang-Yu Zhao, Yi-Han Liu, Xiao-Meng Zhang, Qin Han, Xiao-Yu Wang

**Affiliations:** ^1^ School of Pharmacy, Shandong University of Traditional Chinese Medicine, Jinan, Shandong, China; ^2^ School of Traditional Chinese Medicine, Shandong University of Traditional Chinese Medicine, Jinan, Shandong, China; ^3^ School of Management, Shandong University of Traditional Chinese Medicine, Jinan, Shandong, China

**Keywords:** LncRNA-NEAT1, immune molecules, autoimmune diseases, allergic diseases, cancer, action pathways

## Abstract

NEAT1, a long non-coding RNA (lncRNA), is involved in assembling nuclear paraspeckles that have been found to impact various immune-related diseases, such as autoimmune diseases, allergic diseases, cancer immunity, sepsis, etc. In immune-related diseases, lncRNA-NEAT1 affects the activation, proliferation, and differentiation process of immune cells by interacting with transcription factors and miRNA (MicroRNA) to regulate an expression level in immune-related genes. It can also regulate the apoptosis and autophagy processes of immune cells by regulating inflammatory responses, interacting with apoptosis-related proteins, or regulating the expression of autophagy-related genes, thereby regulating the development of immune-related diseases. In recent years, a large number of researchers have found that the abnormal expression of lncRNA-NEAT1 has a great impact on the onset and progression of immune diseases, such as innate immunity after viral infection and the humoral immunity of T lymphocytes. In this paper, the specific mechanism of action and the function of lncRNA-NEAT1 in different immune-related diseases are sorted out and analyzed, to furnish a theoretical foundation for the study of the mechanism of action of immune cells.

## 1 Introduction

Long non-coding RNAs (lncRNAs) are RNA molecules longer than 200 bases, primarily found in the nucleus and cytoplasm, and they do not encode proteins despite their length. Long non - coding RNAs (lncRNAs) are RNA molecules longer than 200 bases, primarily found in the nucleus and cytoplasm, and they do not encode proteins despite their length. As research advances, scientists have discovered a close correlation between lncRNAs and immune cell function. They are involved in the development of autoimmune diseases by regulating the differentiation and effector function of immune cells (Li and Wu, 2019). Research has revealed that lncRNAs have the potential to effectively regulate immune rejection in organ transplantation, thereby promoting the long-term survival of grafts (Shen and Wang, 2019). Additionally, lncRNAs are significant contributors to the development, activation, proliferation, and differentiation of immune cells (Wang, 2023). By engaging in post-transcriptional regulation, lncRNAs can impact the expression of genes associated with the immune system. This, in turn, allows them to finely tune the inflammatory response, control the proliferation and transformation of immune cells, and influence the intensity and nature of immune responses. It provides an important regulatory basis for the normal conduct of the immune response. This serves as a crucial regulatory framework for orchestrating proper immune responses. Specifically, the long non-coding RNA known as nuclear paraspeckle assembly transcript 1 (NEAT1) holds significant importance in guiding immune processes. According to the latest research, research has revealed that it exerts a function in chronic inflammation, infection, autoimmune diseases, and even immune-related cancers. Therefore, by interfering with the expression or function of lncRNA-NEAT1, new drug treatment strategies may be developed to provide new potential treatment targets for immune-related diseases. This drug development can be carried out by small molecule compound screening, nucleic acid drug design and gene editing technology.

## 2 The biological characteristics and biological functions of lncRNA-NEAT1

**FIGURE 1:**
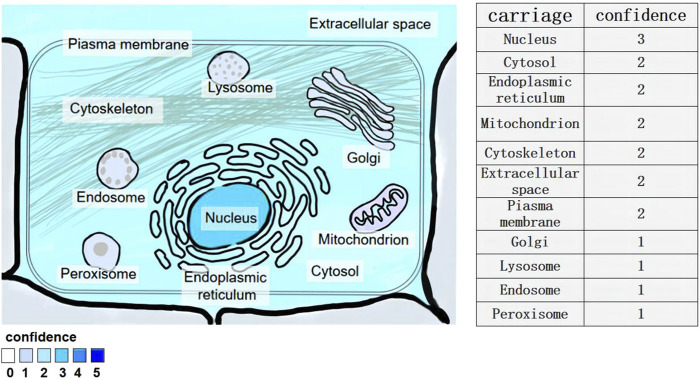
Subcellular location of lncRNA-NEAT1 gene. Human HeLa cells were examined by fluorescence *in situ* hybridization (FISH) and immunofluorescence staining. Fluorescent signals in multiple cell structures were observed via confocal microscopy and scored (3 for strong, 2 for medium, 1 for weak).

LncRNA-NEAT1 is a kind of lncRNA that is abundant in the nucleus. It is a specific subset of lncRNAs and a very important part of the subcellular structure of the nucleus. Situated in the 11q13 region of the human chromosome, the lncRNA-NEAT1 gene is predominantly expressed in various cellular compartments including the endoplasmic reticulum lumen, nuclear envelope, mitochondrial matrix, nucleus, and nuclear pore. It exhibits widespread expression across mammalian cells (Jiang, 2023; Figure 1)

**FIGURE 2:**
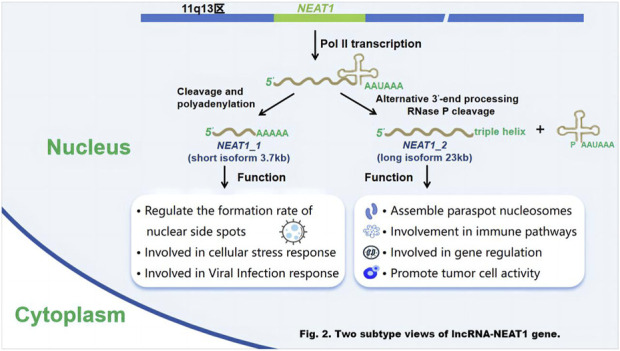
Two subtype views of lncRNA-NEAT1 gene. Schematic diagram of the generation and functions of the two subtypes of the lncRNA - NEAT1 gene. Researchers Sun et al. (2018) used gene - editing techniques (such as the CRISPR/Cas9 system) to edit the NEAT1 gene in human cell lines (such as HEK293T cells), and analyzed transcript changes by RNA sequencing (RNA - seq) to determine the generation pathways of NEAT1_1 and NEAT1_2. Fluorescent labeling combined with confocal microscopy was used to track the localization and functional manifestations of the two subtypes within cells. Functional verification experiments included silencing or overexpressing NEAT1_1 and NEAT1_2, and detecting changes in relevant indicators such as cell stress (Zhang J. et al., 2019), viral infection (Li et al., 2020a) and tumor cell proliferation (Wang Z. et al., 2023).

LncRNA - NEAT1 has two distinct variants: the shorter NEAT1_1 (3.7 Kb) and the longer NEAT1_2 (23 Kb). Both are transcribed from the same starting point and share identical 5′-terminal sequences, but differ in their 3′-terminal regions (Adriaens, 2016) The two are also different in function, and the shorter lncRNA-NEAT1_1 is involved in functions independent of paraspeckles (Naveed et al., 2021). LncRNA-NEAT1_1 is the predominant and well-conserved subtype across various (Isobe et al., 2020) cell types. It exhibits high expression levels in most tissue cells and is typically associated with the structural integrity of nuclear speckles. Under certain circumstances, it can regulate the formation rate of nuclear plaques and has the function of participating in cell stress response and viral infection response. While (Adriaens et al., 2019) the longer NEAT1_2 is responsible for the scaffold, called the gene-regulated nuclear body of the paraspeckles, which is mainly found in the nucleus (Wang and Chen, 2020). LncRNA-NEAT1_2 is typically found in small clusters within specific cell types, such as the tip cells (Kukharsky et al., 2020) of the intestinal epithelium. It has been observed to play an important role in the formation of nuclear structures (Yan et al., 2021) called paraspeckles. It exerts an effective function in the formation of paraspeckles, nuclear bodies, and also aids in the export of mRNA from the nucleus. Moreover, it is recognized as an oncogene that promotes tumor cell proliferation, metastasis, and drug resistance in different types of cancers and solid tumors. At the same time, lncRNA-NEAT1_2 is associated with the modulation of diverse pathological mechanisms, for example, neurodegenerative diseases and inflammation, and functions by participating in immune pathways and gene regulation (Figure 2).

LncRNA - NEAT1 was originally discovered in the process of studying tumors. In the study of prostate cancer (PRAD), an extensive examination of a large group of clinical patient data revealed a notable correlation between the expression levels of lncRNA - NEAT1 and the advancement of prostate adenocarcinoma (PRAD) (Chakravarty et al., 2014). The current study also found that lncRNA-NEAT1 has great potential in the medical diagnosis and prognosis evaluation of cancer (Ma et al., 2016), inflammatory diseases (Yunchun et al., 2023), and neurodegenerative diseases (Shi, 2024). Recent studies have shown that lncRNA-NEAT1 has the following biological functional characteristics: I. Promote and maintain the structural integrity of nuclear paraspots (Santoro et al., 2016), II. It can affect the expression of immune-related genes (Xueyang et al., 2023), III. At the same time, it can also participate in the regulation of inflammatory response (Yunchun et al., 2023), IV. Regulate T cells and macrophages and other immune cells (Huang et al., 2018), VI. Regulate immune cell function, Ⅶ. Function of the regulation of lipid metabolism (Zuo et al., 2021). Through functional characterization, it has been revealed that lncRNA-NEAT1 plays a critical position in modulating immune cell functionality, preserving immune homeostasis, and regulating inflammatory responses. Across a range of immune-related diseases, such as pulmonary fibrosis, sepsis, asthma and allergic rhinitis (AR), the expression level of lncRNA-NEAT1 will change, and this change is closely related to the prognosis of the disease. Hence, lncRNA-NEAT1 shows potential as a diagnostic biomarker for the detection of illness and management of these immune-related (Jin et al., 2020) disorders. lncRNA-NEAT1 also has research value in the treatment of autoimmune diseases. In clinical practice, detecting the level of lncRNA-NEAT1 may be helpful for diagnosis, treatment monitoring and prediction of autoimmune disease progression. Therefore, it has become a valuable target for autoimmune disease research (Zhang 2024). These discoveries enhance our comprehension of lncRNA-NEAT1’s involvement in intricate biological mechanisms, shedding light on its implications in the development of inflammatory and autoimmune conditions. Furthermore, they offer a novel avenue for researching potential treatments for these diseases.

## 3 Immunomodulatory effects of lncRNA-NEAT1

LncRNA has a significant impact on the immune system response and cancer immunotherapy. LncRNA-NEAT1 as one of the most extensively studied lncRNAs, has been found (Zhang P. et al., 2019) in the brain of mice infected with Japanese encephalitis virus and rabies virus and has been confirmed to participate in caspase-1-related typical inflammasome activation, macrophage polarization, DNA-mediated innate immune response and other immune reactions (Morchikh et al., 2017). In recent years, experimental studies on lncRNA-NEAT1 are also deepening its mechanism of action in different immune diseases.

### 3.1 Innate immunity

Innate immunity is also known as non-specific immunity. The effects of lncRNA-NEAT1 on innate immunity can be analyzed from the mechanism of immune molecules and macrophage polarization.

The changes in the expression of lncRNA-NEAT1 in host cells after viral infection can affect the immune molecules. For example, Hantaan virus (HTNV), *in vitro* and *in vivo* experiments have shown (Ma, 2017), HTNV infection can induce host cells to upregulate lncRNA-NEAT1 through the RIG-I-IRF7 signaling pathway. lncRNA-NEAT1 can recruit SFPQ to form para-nuclear spots and release the transcriptional inhibition of RIG-I and DDX60 by SFPQ, thereby increasing RIG-expression I and DDX60 synergistically promote the production of IFN after HTNV infection, thereby inhibiting the replication of HTNV infection and producing antiviral effects. Through the change of lncRNA-NEAT1 expression, the molecules with antiviral effect can be screened and the mechanism of action can be further clarified, which lays a theoretical foundation for the development of new drugs. It has also been shown (Si et al., 2022) that the polarization of macrophages in the tumor microenvironment is regulated by lncRNA-NEAT1,and inhibiting the polarization of M2 cells can silence its expression, furthermore, the proliferation and invasion of tumor cells were improved. Macrophages are important groups in the tumor stroma, which can change their functions through classical activation of M1 type and alternate activation of M2 type, and participate in the development of tumors. M1 macrophages participate in the inflammatory response by regulating the expression of pro-inflammatory factors. In the solid tumor microenvironment, peripheral blood monocytes infiltrate into tumor tissue through blood vessels, and mainly differentiate into M2 type. The results showed that the expression level of lncRNA-NEAT1 in M2 macrophages was significantly increased in tumor tissues, and the expression level of lncRNA-NEAT1 in M2 macrophages was significantly increased, while that in M1 macrophages was significantly decreased, which further suggested that lncRNA-NEAT1 took part in the regulation of M1 and M2 polarization of giant cells. Therefore, understanding and manipulating the role of lncRNA-NEAT1 in regulating immune response may help to enhance existing immunotherapy strategies or develop novel therapeutic approaches to improve the efficacy of cancer treatment.

### 3.2 Cellular immunity

In the immune system, T lymphocyte has a vital impact on the initiation and activation of the immune response. T lymphocyte apoptosis is related to the process of infectious diseases, and can promote the occurrence of autoimmunity and infection.

Experiments have proven that (Zhao and Meng, 2020) lncRNA-NEAT1 showed high expression level in T lymphocytes l in patients with severe pulmonary infection, and miR-495-3p showed low expression level. Further experiments showed that after silencing lncRNA-NEAT1 expression, the apoptosis rate of T lymphocytes was decreased, B-cell lymphoma-2 (Bcl-2) significantly presented high expression level, and bcl-2-associated X protein (Bax) significantly presented low expression level. This suggests that reducing the representation of anti-apoptotic protein Bcl-2 and promoting the representation of apoptotic protein Bax in patients with severe pulmonary infection can increase the apoptosis of T lymphocytes, while silencing the representation of lncRNA-NEAT1 can significantly inhibit the apoptosis of T lymphocytes. Silencing lncRNA-NEAT1 expression can inhibit the apoptosis of T lymphocytes in patients with severe pulmonary infection by up-regulating the expression of miR-495-3p *in vitro* cell experiments, which can provide a theoretical basis for clinical diagnosis and treatment of severe pulmonary infection.

At the same time, T lymphocytes are associated with airway remodeling and inflammatory response in bronchial asthma (BA). Th17/Treg imbalance is involved in the pathological and physiological processes of BA, and the representation of lncRNA-NEAT1 in peripheral blood of children with BA in the acute attack is upregulated (Zheng et al., 2023). It is hypothesized that the expression of lncRNA-NEAT1 is activated during the attack of BA, which can promote the continuous release of inflammatory factors and cytokines and accelerate inflammation (Li et al., 2020b). Moreover, in the acute exacerbation stage, the immune response is abnormally active, and the dysregulation of lncRNA-NEAT1 can aggravate the inflammatory response.

## 4 The role of lncRNA-NEAT1 in immune diseases

LncRNA-NEAT1 plays a critical role in many diseases. It affects the pathogenesis of autoimmune diseases such as Systemic Lupus Erythematosus (SLE), Multiple Sclerosis (MS), and Rheumatoid Arthritis (RA). It plays a vital role in the pathological process of sepsis and allergic diseases. In the field of oncology, lncRNA-NEAT1 is directly involved in the development of various cancers, including Colorectal Cancer (CRC), Breast Cancer (BC), Non-Small Cell Lung Cancer (NSCLC), and Hepatocellular Carcinoma (HCC). Through tumor immune regulation mechanism, new treatment ideas are provided, and it is expected to become a new therapeutic target. In addition, lncRNA-NEAT1 also shows essential effects in the occurrence of gliomas and fibrosis. LncRNA-NEAT1 has potential clinical application value as a critical molecular marker for diagnosis and treatment, and can be used in disease diagnosis or targeted treatment strategies. The following will introduce in detail the research progress of lncRNA-NEAT1 in immune diseases, providing practical support for disease treatment strategies.

### 4.1 Autoimmune disease

**FIGURE 3:**
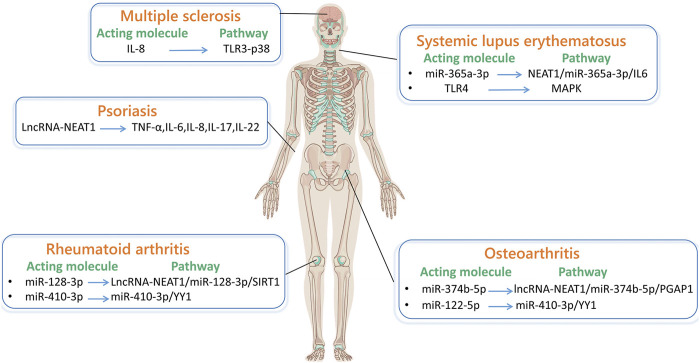
LncRNA-NEAT1 in autoimmune diseases. (By Figdraw.) The main pathways through which lncRNA-NEAT1 acts in systemic lupus erythematosus are NEAT1/miR-365a-3p/IL-6 and MAPK pathways. The pathway through which lncRNA-NEAT1 acts in multiple sclerosis is TLR3-P38. The main pathways through which lncRNA-NEAT1 acts in rheumatoid arthritis are lncRNA-NEAT1/miR-128-3p/SIRT1 and miR-410-3p/YY1. The main pathways through which lncRNA-NEAT1 acts in osteoarthritis are lncRNA-NEAT1/miR-374b-5p/PGAP1, miR-410-3p/YY1. The related factors through which lncRNA-NEAT1 acts in psoriasis are TNF-α, IL-6, IL-8, IL-17, and IL-22.

Autoimmune disease is caused by the immune system wrongly attacking its healthy cells, tissues, or organs. In recent years, some researches showed that lncRNA-NEAT1 plays a crucial character in autoimmune disease. It affects gene expression mediated by nuclear speckles and acts on diseases through signaling pathways and immune cells. The following will introduce in detail the research progress of lncRNA-NEAT1 in autoimmune disease, providing molecular targets for disease treatment strategies (Figure 3).

#### 4.1.1 Systemic lupus erythematosus

SLE is a chronic autoimmune condition characterized by irregular cellular and humoral immune responses. The nosogenesis of SLE is the dysfunction of the autoimmune system, in which various immune cells, cytokines, autoantibodies, receptors (Yongzhen and Wu, 2023), and inflammatory factors participate in the formation of complex cellular and humoral immune processes. A growing body of research shows that (Zhang Xin et al., 2020), lncRNA-NEAT1 is upregulated in immune-related disease, and facilitates the activation of the interferon (IFN) pathway by releasing a B-cell activating factor, thereby contributing to the incidence of SLE.

The phenotype of peripheral blood monocytes in SLE patients has undergone significant changes. By employing real-time fluorescent quantitative polymerase chain reaction technology (Chen et al., 2014) the analysis indicates that lncRNA-NEAT1 shows a higher expression in peripheral leukocytes of individuals with SLE than in the normal population. By calculating IFN score, as is known to all, lncRNA-NEAT1 positively correlates with IFN. That is, lncRNA-NEAT1 affects the pathogenesis of SLE and is involved in the nosogenesis of SLE with some kind of connection to IFN. It has been demonstrated that (Zhang et al., 2016)lncRNA-NEAT1 shows a significantly upregulated expression in monocytes of SLE patients, and the expression shows a positive association with interleukin-6 (IL-6) and IFNγ-induced CXC chemokine ligand 10 (CXCL10). This indicates that cytokines and chemokines of monocytes are the nosogenesis of SLE. In addition, abnormal expression of lncRNA-NEAT1 leads to overactivation of mitogen-activated protein kinase (MAPK) signaling pathway. This overactivation induces overproduction of cytokines and chemokines, resulting in abnormal activation of the immune system and thus triggering SLE. Therefore, lncRNA-NEAT1 is a potential marker for the pathogenesis of SLE.

**FIGURE 4:**
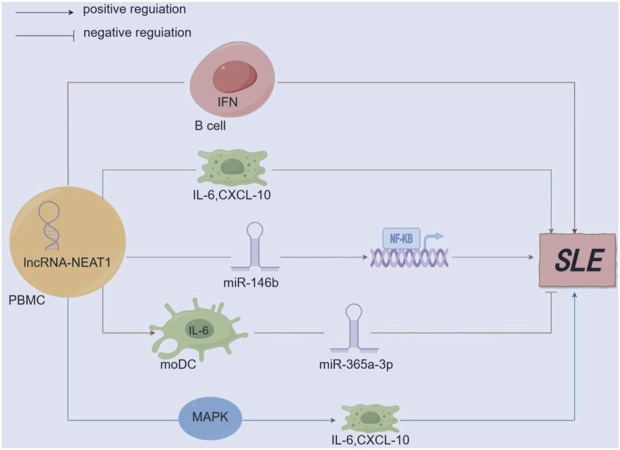
Mechanism diagram of lncRNA-NEAT1 in SLE. (By Figdraw.) lncRNA-NEAT1 is upregulated in immune-related diseases and participates in the pathogenesis of SLE by secreting B-cell activating factor to promote the activation of the interferon (IFN) pathway; lncRNA-NEAT1 positively correlates with interleukin-6 (IL-6) and IFNγ-induced CXC chemokine ligand 10 (CXCL10). Aberrant expression of lncRNA-NEAT1 leads to excessive activation of the MAPK signaling pathway, which induces overproduction of cytokines (IL-6) and chemokines (CXCL10); lncRNA-NEAT1 can promote the secretion of IL-6 in moDCs, while miR-365a-3p mainly functions as a negative regulator; lncRNA-NEAT1 accelerates renal mesangial cell injury by directly targeting miR-146b and activating NF-κB signaling in lupus nephritis.

LncRNA-NEAT1 (Xiang et al., 2023) affects the inflammatory role of dendritic cells (moDC) in SLE. Research findings have reported that lncRNA-NEAT1 can enhance the release of IL-6 in moDC. Conversely, miR-365a-3p (a microRNA that binds to the 3′UTR region of IL-6 and lncRNA-NEAT1) predominantly functions as a suppressor. Namely, the lncRNA-NEAT1/miR-365a-3p/IL-6 axis is associated with the development of SLE disease. Additionally, lncRNA-NEAT1 accelerates mesangial cell injury in lupus nephritis by directly targeting miR-146b, promoting the expression of TRAF6, and activating NF-kB signaling (Zhang L. H. et al., 2020). According to research (Zhu and Luo, 2023), tocilizumab is a monoclonal antibody against the IL-6 receptor and is effective in treating arthritis associated with SLE. At the same time, non-receptor tyrosine kinase (JAK) inhibitors have proven highly valuable in treating SLE-related alopecia and joint pain (Nakayamada and Tanaka, 2022). It can be studied that lncRNA-NEAT1-related JAK inhibitors regulate multiple pathways, providing more options for treatment plans (Figure 4).

#### 4.1.2 Multiple sclerosis

MS is a condition resulting from the immune system attacking and causing inflammation and damage to the protective covering of nerve fibers in the brain and spinal cord, mainly located around the lateral ventricles, accompanied by reactive glial proliferation, and may also have axonal injury. According to research (Zhang Xinyuan et al., 2020), lncRNA-NEAT1 plays a role in neural function. Analysis of transcriptome sequencing of the cerebral cortex of mice with traumatic brain injury model showed significant upregulation of lncRNA-NEAT1 expression, showing that lncRNA-NEAT1 has a specific influence on the nosogenesis of MS.

LncRNA-NEAT1 (Karimi et al., 2022) shows a high expression in peripheral blood mononuclear cells (PBMC) of MS patients. The increased expression of lncRNA-NEAT1 is directly linked to tumor necrosis factor (TNF-α) associated with helper T cell 1 (Th1) and interleukin 17 (IL-17) related to helper T cell 17 (Th17), thereby enhancing vulnerability to MS. By comparing the gender of patients, the discovery reveals a noteworthy rise in the expression level of lncRNA-NEAT1 among female patients, suggesting a notable correlation between lncRNA-NEAT1 expression and a favorable familial background of autoimmune disorders. The expression of lncRNA-NEAT1 is also induced by the translocation of oligodendrocytes and the persistent steroid-responsive protein (stathmin) (Dastmalchi et al., 2018), which shows a high expression in the active brain plaques of patients affected by MS. At the same time, in patients with MS, the transcriptional activation of interleukin 8 (IL-8) (Santoro et al., 2016) may be related to the induction of lncRNA-NEAT1 through the stathmin-activated Toll-like receptor III-mitogen-activated protein kinase 13 (TLR3-p38) pathway. Therefore, lncRNA-NEAT1 plays a crucial character in the pathophysiology of MS.

#### 4.1.3 Rheumatoid arthritis

RA is an autoimmune disease characterized by chronic inflammation, leading to inflammation of synovial tissue in joints. Currently, many studies have shown that many abnormally expressed lncRNAs have been recognized in tissues and cells of RA patients, and the lncRNA-NEAT1 shows a high expression level in the PBMC of individuals with RA. Through research on the binding sites of silent information regulator 1 (SIRT1) and lncRNA-NEAT1 (Zhang Chao et al., 2021), it is found that only miR-128-3p has potential binding sites for both lncRNA-NEAT1 and SIRT1. The lncRNA-NEAT1/miR-128-3p/SIRT1 pathway can regulate the autophagy process of RA cells as well as the secretion of interleukin-6 (IL-6) and matrix metalloproteinase-3 (MMP-3), thereby influencing the pathogenesis of RA. As an mRNA, lncRNA-NEAT1 increases the expression of miR144-3P target gene ROCK2, affecting the proliferation and migration of synovial fibroblasts in RA.

Fibroblast-like synoviocytes (FLS) are the primary cell type responsible for the abnormal proliferation of synovial tissue in RA, which continuously increase, produce large amounts of inflammatory cytokines and matrix metalloproteinases, erode articular cartilage and bone tissue, and play an essential character in the pathophysiology of RA. Hence, gaining knowledge about the fundamental processes of excessively stimulated FLS could aid in recognizing fresh indicators for diagnosis as well as targets for treatment. It has been demonstrated that (Guan, 2021) lncRNA-NEAT1 is upregulated in RA patients, and lncRNA-NEAT1 is overexpressed in human fibroblast-like synoviocytes (HFLS-RA), facilitating the movement, infiltration, and release of inflammatory cytokines in FLS derived from patients with RA. The miRNA miR-410-3p targets explicitly the transcription factor Yin Yang 1 (YY1) in FLS derived from patients with RA. The direct binding of lncRNA-NEAT1 leads to a negative regulation of miR-410-3p expression, while positively controlling YY1, In HFLS-RA, apoptosis is suppressed by the miR-410-3p/YY1 axis. It was detected by Western blot that (Zhou et al., 2021) lncRNA-NEAT1 was upregulated in synovial tissues of RA patients and RA fibroblast-like synoviocytes (RA-FLSs) treated with tumor necrosis factor-**α** (TNF-**α**), while miR-204-5p was downregulated. Namely, knocking down NEAT1 could attenuate TNF-**α**-induced proliferation of RA-FLSs and production of inflammatory cytokines, and promote cell apoptosis by targeting miR-204-5p through the NF-KB pathway. Studies have shown that (Wang M. et al., 2022) miR-221-3p positively correlates with and regulates the level of urokinase-type plasminogen activator receptor (uPAR) in RA-FLS. LncRNA-NEAT1 can predictively target miR-221-3p at three sites, suggesting its potential as a competitive endogenous RNA in RA-FLS. It can act as a rheostat for the miR-221-3p/uPAR axis and downstream JAK signaling, negatively regulating the tumorigenic features and cytokine secretion of RA-FLS.

Additionally, Tetrandrine (Tet) reduces the growth of RA-FLS by declining the expression of lncRNA-NEAT1. This inhibition occurs through the targeting of miR-17-5p (Wang et al., 2020a) and the blocking of the signal transducer and activator of transcription 3 (STAT3). In summary, lncRNA-NEAT1 can act on FLS as a direct target for treating RA.

#### 4.1.4 Osteoarthritis

Osteoarthritis (OA) is a prevalent form of arthritis that affects older individuals, causing persistent pain in the joints. Clinical symptoms of a condition often consist of joint damage, stiffness, and pain. They are typically marked by inflammation of the synovial membrane and deterioration of the cartilage in the affected joints. Furthermore, the contribution of pro-inflammatory cytokines and cartilage cells in the development of OA has been acknowledged. LncRNA is pivotal for the regulation of inflammatory responses and the maintenance of the dynamic balance of chondrocytes, contributing significantly to their overall functionality. The findings of the research demonstrate that there is a decrease in the expression of a specific type of RNA known as lncRNA-NEAT1 in cartilage tissue affected by OA and a noteworthy decline in lncRNA-NEAT1 is noticed (Fu, 2022) in chondrocytes induced by lipopolysaccharide (LPS). The downregulation of lncRNA-NEAT1 can result in a halt at the G1/S phase transition in chondrocytes that have been caused by LPS. In the presence of LPS, the apoptosis rate of lncRNA-NEAT1 small interfering RNA (siRNA)-transfected chondrocytes is higher than that of negative control short interfering RNA (NC siRNA)-transfected cells. In short, decreased levels of the lncRNA-NEAT1 in chondrocytes stimulated with LPS can induce cell death and trigger a cascade of inflammatory reactions. In the mentioned chondrocytes, transfecting lncRNA-NEAT1 siRNA can enhance the expression of miR-374b-5p, ultimately resulting in a similar outcome. MiR-374b-5p targets and regulates the PGAP1 (post-GPI attachment to proteins) gene. When this gene is overexpressed, it can enhance the viability of chondrocytes exposed to LPS. The significance of the lncRNA-NEAT1/miR-374b-5p/PGAP1 pathway in the development of OA highlights its potential as a therapeutic target for clinical intervention.

Both lncRNA-NEAT1 and miR-16-5p are upregulated in OA tissues. LncRNA-NEAT1 can enhance chondrocyte viability and reduce apoptosis (Huang et al., 2023), while miR-16-5p has the opposite effect. However, lncRNA-NEAT1 can specifically bind to miR-16-5p and decrease its expression. The inhibition of miR-16-5p mediated by lncRNA-NEAT1 overexpression can promote chondrocyte proliferation and inhibit apoptosis. LncRNA-NEAT1 is negatively correlated with miR-543 and is lowly expressed in OA cartilage, while PLA2G4A is negatively correlated with miR-543 and is highly expressed in OA cartilage. In OA chondrocytes, overexpressed lncRNA-NEAT1 upregulates the expression of interleukin (IL)-6 and IL-8 (Li D. et al., 2020), but this effect of overexpressed lncRNA-NEAT1 is reversed by miR-543 mimics. Overexpression of PLA2G4A has the opposite effect on miR-543 mimics in OA chondrocytes. In summary, lncRNA-NEAT1 can sponge miR-543 to induce PLA2G4A expression, inhibiting chondrocyte proliferation and promoting apoptosis.

LncRNA-NEAT1 is a target of miR-146a-3p, and miR-146a-3p is a target gene of tyrosine kinase receptor B (TrkB). The literature reports (Xiao et al., 2021) that TrkB is over-expressed in OA and is correlated with chondrocytes stimulated by proinflammatory cytokines (IL-1β). Knocking down TrkB can improve the biological function of chondrocytes stimulated by IL-1β. Therefore, knocking down the expression of lncRNA-NEAT1 enhances the proliferative, migratory, and invasive abilities of chondrocytes, inhibits cell apoptosis, and reverses the effect of miR-146a-3p inhibitors on chondrocytes. Co-culturing overexpressed lncRNA-NEAT1 from OA patient cartilage tissue with human bone marrow mesenchymal stem cell-derived extracellular vesicles (BMSC-EVs) (Ning et al., 2021) revealed that lncRNA-NEAT1 can be transferred from BMSC-EVs to chondrocytes, resulting in high expression of miR-122-5p and low expression of the highly conserved cellular stress-induced protein (Sesn2). LncRNA-NEAT1 binds to miR-122-5p to restrict miR-122-5p expression targeting Sesn2 and activate the transcription factor (Nrf2) pathway. Meanwhile, BMSC-EVs, miR-122-5p downregulation, or Sesn2 overexpression delivered by lncRNA-NEAT1 induce chondrocyte proliferation and autophagy. In summary, BMSC-EVs containing lncRNA-NEAT1 prevent OA by activating the Sesn2/Nrf2 axis through binding to miR-122-5p. Overall, knocking down lncRNA-NEAT1 can regulate chondrocyte proliferation and apoptosis in OA, and the development of lncRNA-NEAT1-based targeted drugs plays a crucial character in the treatment of OA.

#### 4.1.5 Psoriasis

Psoriasis is an inflammatory skin disease caused by autoimmunity under a polygenic genetic background, which is extremely prone to recurrence and challenging to cure. Research results indicate (Zhang and Jin, 2022) that the expression level of SCL in peripheral blood T cells and lncRNA-NEAT1 is reduced in psoriasis, thereby affecting the pathogenesis of psoriasis. Downregulation of lncRNA-NEAT1 in psoriasis can regulate hemoglobin synthesis, affect the development and proliferation of *streptococcus*, stimulate the activation of T lymphocytes, and participate in the occurrence and advancement of psoriasis lesions.

LncRNA-NEAT1 is positively correlated with the activity of psoriasis (Xinhua et al., 2013), and its expression level in psoriasis skin tissue is higher than that in normal skin tissue. Simultaneously, the expression level of lncRNA-NEAT1 in lesional skin tissue is positively correlated with the levels of TNF-α, IL-6, IL-8, IL-17, and interleukin 22 (IL-22), indicating that lncRNA-NEAT1 enhances disease activity by releasing inflammatory cytokines (IL-6, IL-8, etc.) through some inflammatory pathways (such as MAPK). Compared to the group that was not influenced (Tang et al., 2023a), the relative expression levels of lncRNA-NEAT1 and STAT3 mRNA are increased in psoriasis tissue, while the miR-485-5p mRNA is reduced. One target of lncRNA-NEAT1 is miR-485-5p, and STAT3 is a target gene of miR-485-5p. It is inferred that lncRNA-NEAT1 can control the expression of STAT3 by acting as a competitive endogenous RNA (ceRNA) to adsorb miR-485-5p, which is one of the essential mechanisms for the occurrence of psoriasis. In summary, knocking down lncRNA-NEAT1 can inhibit T lymphocyte activation and interleukin production, improve psoriasis symptoms, and provide possibilities for experimental research on lncRNA-NEAT1 inhibitors.

LncRNA-NEAT1 and Galectin-7 are lowly expressed in psoriasis patients, while miR-3194-5p is highly expressed. Paeoniflorin inhibits the proliferation and migration of psoriasis HaCat cells by upregulating the expression of lncRNA-NEAT1 and Galectin-7 (Tang et al., 2023b). Paeoniflorin controls the proliferation and migration of psoriasis HaCat cells through the NEAT1/miR-3194-5p/Galectin-7 axis. The impact of specific components in paeoniflorin on the NEAT1/miR-3194-5p/Galectin-7 axis can be studied to provide new therapeutic insights.

#### 4.1.6 Other autoimmune diseases

Autoimmune disease is a state of illness caused by the immune system of the body responding to its components. LncRNA-NEAT1 is related to regulating the immune system and plays a key character in autoimmune diseases (Wang D. et al., 2022). In addition to the diseases discussed above, there are many other autoimmune diseases worthy of our research. Qin et al. (2021) and his colleagues found that lncRNA-NEAT1 is downregulated in cardiomyocytes and heart tissue of patients with diabetes mellitus (DM). The combined use of Dendrobium mixture and metformin (Met) upregulates lncRNA-NEAT1 in DM patients, which can effectively inhibit cardiomyocyte apoptosis and improve heart tissue morphology. Dendrobium mixture and Met can be used in combination to inhibit diabetes. Pi et al. (2021) found through experiments that lncRNA-NEAT1 is upregulated in tubular tissue of membranous nephropathy (MN), and overexpression of lncRNA-NEAT1 can induce apoptosis and reduce the proliferation of tubular epithelial cells. Meanwhile, overexpression of lncRNA-NEAT1 significantly increases the mRNA and protein levels of vancomycin (Noxa), but only the interference of BH3 protein Noxa can alleviate tubular epithelial cell apoptosis. Therefore, inhibiting Noxa-mediated anti-apoptotic activity plays a vital character in the mechanism by which lncRNA-NEAT1 promotes the advancement of MN by inducing apoptosis. Different types of virus infections have different consequences for the body. Rahni et al. (2023) and his colleagues found that lncRNA-NEAT1 is upregulated in moderate to severe coronavirus disease (COVID-19), and lncRNA-NEAT1 triggers the synthesis of IL-6, a key contributor to the body’s immune defense against SARS-CoV-2. It can be seen that lncRNA-NEAT1 plays a role in the advancement is of many autoimmune diseases, however, main pathways and roles it participates in still need further exploration to provide treatment options for more autoimmune diseases.

### 4.2 Anaphylaxis

In recent years, an increasing number of studies have focused on the role of lncRNA-NEAT1 in allergic diseases, especially in the occurrence and development of asthma, allergic rhinitis (AR), and atopic dermatitis.

Asthma is a chronic inflammatory airway disease characterized by airway hyperresponsiveness and airflow limitation (Varricchi et al., 2022). Imbalance in immune responses, particularly the abnormal activation of Th1 and Th2 cells is the direct cause of asthma and is associated with the aberrant expression of lncRNAs. Significantly elevated levels of lncRNA-NEAT1 and GATA3 have been found in peripheral blood CD4^+^ T cells of children with asthma (Yan et al., 2023). Knockdown of lncRNA-NEAT1 or GATA3 markedly reduced Th2-associated cytokines (such as IL-4, IL-5, and IL-13), and T-cell proliferation, which in turn controlled asthma-related airway inflammation. Mechanistically, lncRNA-NEAT1 participates in the regulation of immune responses by interacting with the NF-κB signaling pathway, which is pivotal for the activation of Th2 cells and the secretion of Th2 cytokines. Duan et al. (2021) demonstrated that silencing of lncRNA-NEAT1 significantly decreased the levels of TNF-α, IL-4, and IL-13 while upregulating IL-10 expression, inhibiting cell proliferation, and promoting apoptosis. IncRNA-NEAT1 may become a potential therapeutic target for asthma. Additionally, airway smooth muscle cells (ASMCs) play a significant role in the pathogenesis of asthma. A study by Wang et al. (2021b) found that inhibits platelet-derived growth factor (PDGF)-induced human airway smooth muscle cell proliferation by targeting SLC26A2 via miR-9-5p, activating the PI3K-AKT signaling pathway, suppressing inflammatory factor production, and influencing airway smooth muscle contraction and relaxation.

Building on previous studies, a significant association between lncRNA-NEAT1 and allergic rhinitis (AR) has been identified (Miller et al., 2021). It has been demonstrated that lncRNA-NEAT1 is overexpressed in individuals with AR, and its target microRNA, miR-125a, regulates immune and inflammatory processes associated with AR through the JAK-STAT signaling pathway. In children with AR, dysregulation of circulating lncRNA-NEAT1 and miR-125a expression correlates with Th2 cells and symptom severity. Another study by Wang T. et al. (2021) revealed that lncRNA-NEAT1 interacts with miR-511 in nasal epithelial cells, activating the MAPK signaling pathway, regulating cell differentiation, inflammation, and immune responses, and directly affecting mucus secretion and cell apoptosis. In summary, lncRNA-NEAT1 plays a crucial role in the occurrence, development, and inflammation of AR, (Wang R. et al., 2021), showing potential as a diagnostic and therapeutic target for AR.

### 4.3 Sepsis

LncRNA-NEAT1 plays a crucial role in the pathogenesis of sepsis, a severe condition resulting from infection-induced systemic inflammation (Almalki, 2024). It is significantly upregulated in sepsis patients and contributes to disease progression by regulating inflammatory factors (Wei et al., 2020) Lipopolysaccharide (LPS)-induced inflammation and apoptosis are mediated through the NEAT1/miR-370-3p axis (Wu et al., 2020), playing a central role in the initiation of sepsis.

Furthermore, lncRNA-NEAT1 promotes sepsis by influencing the migration and proliferation of inflammatory cells. It targets MiR-27a, which inhibits PTEN,and the silencing of miR-27a reverses the effects of lncRNA-NEAT1 silencing, impeding cell proliferation (Lv et al., 2021) This indicates that lncRNA-NEAT1 may regulate cellular processes critical to the immune response and inflammatory progression in sepsis.

Further investigations have shown that lncRNA-NEAT1 plays a pivotal role in LPS-induced cardiomyocyte injury (Wang et al., 2019) by modulating various signaling pathways. Knockdown of lncRNA-NEAT1 blocking the TLR2/NF-κB signaling pathway, mitigating LPS-induced cardiomyocyte injury (Feng et al., 2020). Additionally, lncRNA-NEAT1 interacts with the PI3K/AKT signaling pathway, promoting cellular proliferation and survival, which further exacerbates sepsis progression. By modulating both NF-κB and PI3K/AKT pathways (Pan T. et al., 2022), lncRNA-NEAT1 amplifies inflammatory responses, promotes immune cell activation, and impairs cellular function, highlighting its potential as a diagnostic and therapeutic target in sepsis.

Overall, lncRNA-NEAT1 influences inflammatory factor expression, mediates inflammatory cell proliferation, and contributes to the pathogenesis of sepsis by activating NF-κB, PI3K/AKT, and other signaling pathways, making it a promising candidate for clinical intervention.

### 4.4 Glioma

Glioma, also known as glioblastoma, is a tumor that occurs in the neuroectodermal layer, also known as neuroectodermal tumor or neuroepithelial tumor. Treatments of glioma (Liu and Wang, 2022) in addition to surgery, radiotherapy and chemotherapy, immunotherapy has become the fourth treatment mode, this is mainly related to the immunosuppressive microenvironment in which tumor cells are located. As demonstrated by previous studies, lncRNA-NEAT1 is involved in several pathways in the formation and development of gliomas and makes a difference to their progression.

Na +/H + exchanger 5 (NHE5) is a sodium-hydrogen exchanger protein, which is mainly expressed in glioma cell line C6. Cao (Cao et al., 2022) finds that there is an antagonistic relationship between miR-22-3p and NHE5, and lncRNA-NEAT1 can negatively regulate miR-22-3p and increase NHE5 expression, thereby promoting the spread and invasion of glioma cells In the glioma cell line C6. A study (Zhang, 2022) has shown that glioma cell lines and samples have higher expression levels of lncRNA-NEAT1, which may function as an oncogene to encourage glioma cell growth both *in vivo* as well as *in vitro*. Downregulation of lncRNA-NEAT1 expression can prevent glioma cells from proliferating, and the mechanism is that after down-regulating the level of lncRNA-NEAT1 expression, the G0/G1 phase of the cell cycle of glioma U251 and LN229 cells is significantly accumulated, on the contrary, the proportion of cells in S phase is reduced. Furthermore, the expression of potassium channel multimer domain protein 20 antibody (KCTD20) and lncRNA-NEAT1 are highly linked in the clinical sample experiment, the decreased expression of lncRNA-NEAT1 and KCTD20 can also lead to the inhibition of the expression levels of cyclin proteins (Cyclin-Dependent Kinases4 and Cyclin D1) and apoptosis-related protein Bcl-2, while the level of pro-apoptotic protein Bax is upregulated, which causes the glioma cell proliferation efficiency to decline and increases the percentage of apoptosis to a certain extent. The opposite is the downregulation of miR-324-5p expression can upregulate the proliferation rate of glioma cells and reduce apoptosis. This study further illustrates the relationship between lncRNA-NEAT1-miR-324-5p-KCTD20: Both lncRNA-NEAT1 and KCTD20 have the same binding site with miR-324-5p, and lncRNA-NEAT1 effectively controls KCTD20 expression by competitively inhibiting miR-324-5p. This finding indicates that lncRNA-NEAT1-miR-324-5p-KCTD20 controlling pathway may be an effective medicinal target for the glioma treatment, and lncRNA-NEAT1 can as a sign of prognosis for glioma patients. In a glioma experimental study (Yu et al., 2021), it has been found that lncRNA-NEAT1 is highly expressed in glioma tissues and cells, while miR-185-5p is lowly expressed, and there is a negative correlation between both of them. When lncRNA-NEAT1 is knocked down, the expression of proliferation antigen Ki-67, DNA methyltransferase 1 (DNMT1) and mammalian target of rapamycin (mTOR) are decreased, but the expression of miR-185-5p is increased, indicating that lncRNA-NEAT1 is a ceRNA of miR-185-5p, which can promote the expression of DNMT1 and activate mTOR signal, thus stopping apoptosis and encouraging glioma metastasis, growth, and shifting between epithelial and mesenchymal cells. A recent study (Liang et al., 2022) has been found that lncRNA-NEAT1 knockdown significantly reduces glioma cell proliferation and glycolysis, and lncRNA-NEAT1 increases the protein level of PGK1. This study further clarifies the particular area of connection between lncRNA-NEAT1 and PGK1: lncRNA-NEAT1’s hairpin A engaged with PGK1’s M1 domain, facilitating cell growth and glycolysis. These findsings indicate that lncRNA-NEAT1 and its related molecular pathways may become potential targets for glioma treatment and present fresh approaches to dealing with the glioma. In the study (Li et al., 2021) of Li et al.,lncRNA-NEAT1 can act as a molecular sponge to regulate miR-98-5p, and can directly target basicleucine zipper and W2do mains1(BZW1), thereby regulating the proliferation of glioblastoma cells. The lncRNA-NEAT1/miR-98-5p/BZW1 axis plays an important role in glioblastoma, so targeted therapies to this axis can be explored. Bi et al. (2020) have identified let-7g-5p, a downstream target of lncRNA-NEAT1 in glioma, and is negatively regulated by it, and the restoration of let-7g-5p inhibits proliferation, migration, and invasion to prevent tumor progression. As a direct target of let-7g-5p, Mitogen-Activated Protein Kinase 1 (MAP3K1) is positively regulated by lncRNA-NEAT1 and participates in the regulation of lncRNA-NEAT1 on the behavior of glioblastoma stem cells (GSC). The results indicate that lncRNA-NEAT1 promotes GSC progression through the lncRNA-NEAT1/let-7g-5p/MAP3K1 axis. A study (Liu et al., 2020) shows that microRNA-92b (miR-92b) inhibits glioma cell apoptosis by downregulating Dickkopf WNT Signaling Pathway Inhibitor 3 (DKK3). Overexpression of lncRNA-NEAT1 can downregulate miR-92b and subsequently upregulate DKK3 to inhibit glioma cell proliferation and promote apoptosis. In a new study (Zakutansky et al., 2024), researchers have discovered differential dysregulation of lncRNA-NEAT1 isoforms in patient-derived glioblastoma multiforme cells. CRISPR-Cas9-mediated PAS deletion can reduce lncRNA-NEAT1_1 and increase lncRNA- NEAT1_2, thereby enhancing the formation of nuclear paraspeckles in human glioma cells. The forced increase of lncRNA-NEAT1_2 after lncRNA-NEAT1 PAS deletion is responsible for driving glioma cell migration and promoting cell interaction and regulateing gene expression of migration. This study discovers a novel mechanism that regulates lncRNA-NEAT1 isoforms and their functional impact on the glioma transcriptome, which affects pathological pathways of glioma represented by migration.

At present, the primary area of research for the glioma treatment is on how lncRNA-NEAT1 binds to particular miRNAs to influence the miRNA target genes. By contrast, there are relatively few researches on the mechanism of lncRNA-NEAT1 and Phosphoglycerate kinase 1(PGK1) in glioma. PGK1 has complex and multiple functions in cancer occurrence and tumor progression. For example, PGK1 (He et al., 2022) can promote renal clear cell carcinoma. The mechanism of action of lncRNA-NEAT1 and PGK1 provides more ideas for the creation of new drugs for the treatment of glioma.

### 4.5 Fibrosis

Fibrosis refers to the excessive process of cell proliferation and collagen synthesis after some tissues or organs are stimulated or damaged, which results in normal cells and tissues are replaced or wrapped to form a large number of fibrous tissues. It usually occurs in livers, lungs, hearts, kidneys and other tissues as well as organs. LncRNA-NEAT1 has been confirmed to be involved in inflammatory processes such as chemokine regulation, cytokine production and inflammasome activation, and has a significant impact on the occurrence and development of fibrosis in different tissues as well as organs.

The activation of hepatic stellate cell (HSC) is the central link in the course of liver fibrosis progression. Autophagy as a metabolic process in cells, that may fuel HSC activation by breaking down lipid droplets, which can encourage the occurrence and development of liver fibrosis. It has been found (Kong, 2020) that insulin-like growth factor binding protein-related protein 1 (IGFBPrP1) and transforming growth factor beta 1 (TGFβ1) regulates each other and jointly increase the onset and progression of liver fibrosis. By this time, IGFBPrP1 can also promote the autophagy and activation of HSC, thereby participating in the progression of liver fibrosis. This study further reveals the mechanism of IGFBPrP1 promoting autophagy and activation of HSC: IGFBPrP1 can promote the upregulation of lncRNA-NEAT1 expression in mouse HSC. lncRNA-NEAT1 competitively bounds to miR-29b and reduces the inhibitory effect of miR-29b on autophagy related 9 homolog A (Atg9a), thereby up-regulating Atg9a expression and exerting the important role of Atg9a in the initiation of autophagy. IGFBPrP1 regulates the lncRNA-NEAT1/miR-29b/Atg9a pathway, which is an important mechanism for the formation of liver fibrosis. Tan et al. (2023) study the effect of lncRNA-NEAT1 on myocardial fibrosis in rats with atrial fibrillation (AF), they find that lncRNA-NEAT1 shows a higher expression level in rat left atrial tissue, while miR-27b-3p has a lower expression level. Downregulation of endogenous lncRNA-NEAT1 can significantly alleviate AF in rats, but the inhibition of miR-27b-3p can reverse the AF effect of lncRNA-NEAT1 downregulation in rats. It is suggested that there is a new lncRNA-NEAT1/miR-27b-3p axis regulatory factor in AF rats, lncRNA-NEAT1 can be used as a ceRNA to promote the expression of Specificity Protein 1 (SP1) by inhibiting the expression of miR-27b-3p, thus playing a role in regulating SP1 and providing new ideas for molecular targeted therapy of AF myocardial fibrosis. Furthermore,in a study of myocardial fibrosis (Zhang X. et al., 2021), it is found that the activation of NOD-like receptor thermal protein domain associated protein 3 inflammasome (NLRP3 inflammasome) is suppressed by lncRNA-NEAT1, thereby down-regulating the NLRP3 inflammasome-induced pyroptosis of various cells in myocardial tissue and slowing down the process of myocardial fibrosis. The relationship between lncRNA-NEAT1 and NLRP3 inflammasome may be exploited as a key target to control the onset and progression of myocardial fibrosishas and generates suggestions for a novel therapeutic drug development.

In hypoxia-induced pulmonary fibrosis, a study (Xu, 2022) has been found that in pulmonary fibrosis tissues as well as cells, lncRNA-NEAT1 is highly expressed, which promotes the apoptosis of alveolar epithelial cells and upregulates the expression of pulmonary fibrosis index protein, while miR-29a is lowly expressed. When lncRNA NEAT1 is knocked out, it can inhibit hypoxia-induced apoptosis of alveolar epithelial cells and reduce the level of pulmonary fibrosis index protein. However, inhibition of miR-29a can reverse this effect and promote the process of pulmonary fibrosis. This study further confirmes that lncRNA-NEAT1 promotes hypoxia-induced pulmonary fibrosis by targeting inhibition of miR-29a, offering a fresh target for the therapy of hypoxia-induced pulmonary fibrosis.

### 4.6 The role of lncRNA-NEAT1 in cancer

In the development and progression of various cancer types, the pivotal role of lncRNA expression is evident. As revealed by recent investigations, lncRNA shows a high expression in colorectal cancer (CRC), breast cancer (BC), lung adenocarcinoma (LC), hepatocellular carcinoma (HCC), and prostate adenocarcinoma (PRAD) (Hao et al., 2023). Using the ENCORI online database, researchers detected hub mRNAs, miRNAs, and lncRNAs that significantly interact with lncRNA-NEAT1. They found that the RNA and protein interaction network of lncRNA-NEAT1 can regulate its expression levels and directly or indirectly evaluate the expression of key hub RNAs in this network (Azadeh et al., 2022). Therefore, lncRNA-NEAT1 serves as a critical molecular regulator of various biological processes and disease conditions.

**FIGURE 5:**
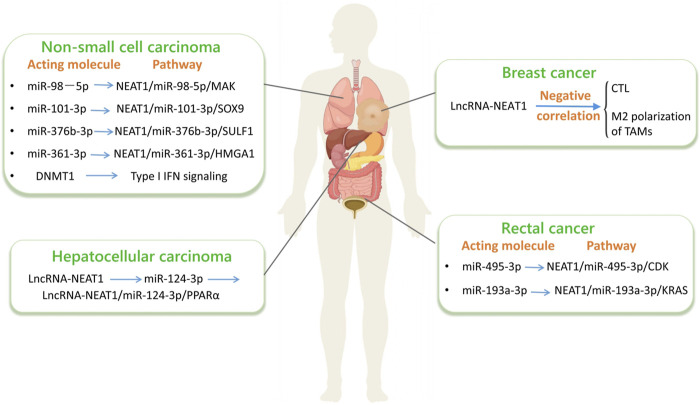
LncRNA-NEAT1 in cancer. (By Figdraw.) The interactions among non - coding RNAs (such as lncRNA and miRNA) and their related signaling pathways in different types of cancers, including non - small - cell lung cancer, hepatocellular carcinoma and colorectal cancer. Specifically, it details the key miRNAs, lncRNAs, their regulated target genes and signaling pathways in each type of cancer, which is helpful for understanding the molecular mechanisms of these cancers.

lncRNA-NEAT1 is considered a regulatory factor involved in cell differentiation, migration, innate immune response, and the progression of cancer. In cancer, it functions as either an oncogenic driver or a tumor-suppressive gene. The deregulation of lncRNA-NEAT1 can affect various cellular processes. In recent years, researchers have elucidated the mechanism of action of this gene in various cancers. Studies on the lncRNA-NEAT1 signaling pathway have shown that it is an oncogene in cancers such as CRC and BC. Therefore, for various cancers, targeting lncRNA-NEAT1 holds promising potential as a therapeutic strategy and lncRNA-NEAT1 can be utilized as a diagnostic tool for late-stage malignancies. LncRNA-NEAT1 also has potential implications in tumor immune modulation. The maturation and function of dendritic cells (DCs) can be influenced by it, thereby influencing T-cell activation and regulation. Additionally, the involvement of lncRNA-NEAT1 is observed in regulating macrophage polarization and influencing the expression of cytokines and chemokines, which are important factors in tumor immune responses. Understanding the specific mechanism of action of lncRNA-NEAT1 in tumor immune regulation can provide clues for the development of novel immunotherapy strategies. As a key regulatory molecule in the tumor microenvironment, lncRNA-NEAT1 is increasingly important in improving tumor treatment outcomes and precision medicine strategies (Figure 5).

#### 4.6.1 Colorectal cancer

CRC stands out as a prevalent malignancy, characterized by elevated incidences and substantial mortality rates. It ranks fifth among all tumors in China, commencing from the inner linings of the appendix, colon, and rectum (Ottaiano et al., 2022). Based on recent research findings, there is an upregulation of lncRNA-NEAT1 and lncRNA-NEAT1 has a carcinogenic effect in CRC (Xiong et al., 2017; Peng et al., 2017; Wu et al., 2015). In patients with CRC, the expression of lncRNA-NEAT1 shows a high expression when compared to the expression levels observed in normal tissues.

Immunohistochemical detection has revealed a substantial upregulation in the expression of lncRNA-NEAT1 in CRC based on numerous studies (Jiang et al., 2020). Suppression of lncRNA-NEAT1 hampers the proliferation, cell cycle progression, migration, and invasive capabilities of CRC cells. Also, by engaging with miR-495-3p, it facilitates tumor cell apoptosis to inhibit the expression of cell division protein kinase 6 (CDK6). However, lncRNA - NEAT1 is capable of interacting with miR - 495 - 3p, thereby influencing the Wnt signaling pathway. MiR - 495 - 3p functions as a targeting molecule for the Wnt inhibitor WIF1. Specifically, when the expression level of miR - 495 - 3p is enhanced, it will lead to a decrease in the protein abundance of WIF1. Consequently, this will result in an increase in the protein levels of β - catenin and c - Myc. The elevated protein levels of β - catenin and c - Myc play crucial roles in promoting the proliferation, migration, and invasion of CRC cells, while simultaneously inhibiting the process of cell apoptosis. In clinical practice, to evaluate the levels of lncRNA-NEAT1 expression, researchers employed real-time fluorescence quantitative PCR on 239 paired CRC samples and adjacent histologically normal tissues. Considering that lncRNA-NEAT1 is comprised of NEAT1_1 and NEAT1_2, experiments were designed and validated using two primer pairs to quantitatively detect lncRNA-NEAT1 isoforms by real-time PCR. A single primer pair had the ability to simultaneously detect and identify both NEAT1_1 and NEAT1_2 (collectively referred to as total NEAT1), whereas the second primer pair specifically targeted NEAT1_2 alone (Zeng et al., 2014; Zhang et al., 2013). Real-time fluorescence quantitative PCR results showed that the levels of lncRNA-NEAT1 expression in CRC were higher than those in normal specimens. According to experimental data, compared with the corresponding normal counterparts, the expression level of lncRNA-NEAT1 was significantly upregulated in 72.0% of CRC specimens (Li et al., 2015). Therefore, we can consider the upregulation of lncRNA-NEAT1 as an informative predictor for the diagnosis and prognosis of CRC.

LncRNA-NEAT1 is also associated with 5-Fluorouracil (5-FU) resistance in CRC (Liruina and Liningning, 2023). Wang et al. (2020b) found that the deletion of lncRNA-NEAT1 promotes the sensitivity to 5-FU, enhances cell apoptosis, and inhibits CRC cell invasion as shown in their study. Additionally, we found that lncRNA-NEAT1 can affect the interaction between various immune cells and CRC tumor cells by regulating apoptosis pathways, providing new ideas for tumor immune regulation mechanisms in CRC tumor treatment. Furthermore, it has been found that lncRNA-NEAT1 affects CRC drug resistance by regulating CRC stemness. Downregulation of lncRNA-NEAT1 reduces the expression of stemness factors, mainly by affecting chromatin remodeling, leading to increased acetylation levels in the ALDH1 and c-Myc protein promoter regions, thereby enhancing CRC cell stemness and promoting 5-FU resistance (Zhuy and Yuanz, 2020). Zhu Zhouting also found (Zhu, 2018) that lncRNA-NEAT1 can specifically inhibit the expression of miR-193a-3p, thereby indirectly inhibiting miR-193a-3p′s post-transcriptional regulation of KRAS mRNA, leading to increased KRAS protein expression. Therefore, we found that lncRNA-NEAT1 affects CRC development through the miR-193a-3p/KRAS pathway, providing a potential target for CRC treatment.

Investigation of lncRNA-NEAT1 expression levels in patients with CRC, which uses bioinformatics analysis, as demonstrated in various other studies. For qRT-PCR, Western blot, and immunohistochemistry assays, clinical samples such as peripheral blood and CRC tissues were gathered to validate that lncRNA-NEAT1 can knock down proliferation and migration of CRC cells by mediating immune inflammatory responses (Chen et al. 2022). It was concluded that the abnormal overexpression of lncRNA-NEAT1 in CRC tissues leads to poor prognosis. Therefore, mechanistically, Immune response activation induced by lncRNA-NEAT1 enhances the growth and movement of CRC cells, providing new insights into inhibiting cancer cell metastasis in immunological aspects and constructing tumor immune regulation mechanisms.

#### 4.6.2 Breast carcinoma

Among women, BC stands as the prevailing form of cancer. Within the realm of malignancies, lncRNAs have garnered recognition for their critical role in governing gene expression.

LncRNA-NEAT1 is highly sensitive to BC. Using real-time fluorescent quantitative PCR, the researchers assessed the expression levels of lncRNA-NEAT1 and lncRNA-XIST in BC patients (Bantug et al., 2018). The results demonstrated a remarkable increase in lncRNA expression in BC patients compared to other groups. Compared with lncRNA-XIST, lncRNA-NEAT1 has better sensitivity in detecting BC with high-risk factors. Therefore, the detection of lncRNA-NEAT1 in body fluids is of great significance for detecting BC patients with high-risk factors.

Erik Knutsen and colleagues conducted a study (Knutsen et al., 2022)^,^ they discovered that a noticeable correlation was observed in relation to the expression level of NEAT1-2 and the grade of breast cancer tumors, as well as the presence of human epidermal growth factor receptor 2 (HER2)-positive BC samples. HER2-positive BC patients shows a higher expression in NEAT1-2, which is associated with more aggressive tumor characteristics. NEAT1-2 therapy may help in immunotherapy. However, overexpression of HER2 can activate the PI3K/Akt/mTOR signaling pathway and promote the proliferation and survival of breast cancer cells. lncRNA-NEAT1 may regulate HER2 signaling pathway by affecting the expression or activity of HER2 and promote the progression of breast cancer. In addition, the expression of NEAT1-2 in human breast tissue increases during lactation. Previous studies have shown that (Sangsuwan et al., 2020) In adult mouse tissues, NEAT1-1 shows expression in various cell types. However, the epithelial layer of digestive tissue is the main site of NEAT1-2 expression. Therefore, Regulating various biological processes and disease states, this lncRNA plays a pivotal role as a key molecule. Variations in lncRNA-NEAT1 expression can result in various diseases, including BC.

Tumor immunity is a complex and dynamic process involving the interaction between tumor cells and immune cells, which is particularly critical in BC development. BC cells and cytotoxic T lymphocytes (CTLs) in the immune system share numerous metabolic characteristics (Chen et al., 2020), such as high glucose demand, leading to intense metabolic competition in the tumor microenvironment and causing resource scarcity that affects immune cell function. Studies have shown that lactate produced by tumor cells can inhibit the activity of CTLs, reduce their proliferation and cytokine production, and cause a 50% decrease in their cytotoxicity, thereby promoting tumor growth and evading immune surveillance. Hence, the development and progression of BC are greatly influenced by the tumor microenvironment, highlighting its significance in devising effective treatment strategies, particularly immunotherapy approaches. Targeting the tumor microenvironment also holds considerable importance in the realm of cancer treatment.

In addition, we found that the role of lncRNA-NEAT1 in BC treatment can also be revealed through immune regulatory mechanisms. To explore the expression and mechanism of lncRNA-NEAT1 in BC tumor-associated macrophages (TAMs), Si Gang and Si Zhen conducted a recent study (Swellam et al., 2021), collecting tumor tissue samples from 30 patients with BC. Through the utilization of density gradient centrifugation employing dextran and metrizamide, coupled with RT-PCR detection techniques, researchers observed a substantial increase in the expression of lncRNA-NEAT1 in tumor-associated macrophages (TAMs) compared to macrophages. Furthermore, upon silencing lncRNA-NEAT1 expression, the proliferation and invasion capabilities of BC cells were hindered, attributed to the inhibition of TAM polarization towards the M2 phenotype. Hence, immune cells such as macrophages have the ability to regulate the expression of lncRNA-NEAT1, offering potential therapeutic strategies for the treatment of BC. It was further found that lncRNA-NEAT1 can be controlled by immune regulation to provide new ideas and methods for the development of BC immunotherapy.

#### 4.6.3 Lung cancer

Globally affecting society, LC is a malignant disease of grave consequences. According to the GLOBOCAN 2020 cancer statistics, LC accounted for 2,206,771 new cases and resulted in the unfortunate loss of 1,796,144 lives. NSCLC accounts for 80% of LC.

LncRNA is closely related to NSCLC. Compared with normal human lung epithelial BES-2B cells, NSCLC cell lines exhibit upregulation of lncRNA-NEAT1. Therefore, The progression of NSCLC cells can be impeded by suppressing lncRNA-NEAT1. LncRNA-NEAT1 upregulates the expression of MAPK, sex-determining region Y box 9 (SOX9), and sulfatase 1 (SULF1) by adsorbing miR-376b-3p, miR-101-3p, and miR-98-5p, respectively, promoting cancer progression. Therefore, as a prognostic biomarker, lncRNA-NEAT1 shows potential as a diagnostic tool and therapeutic target in the management of NSCLC. The activation of the TLR4/NF - κB signaling pathway has a close correlation with tumor invasion, metastasis, and immune evasion in NSCLC. Long non - coding RNA - NEAT1 is likely to regulate the TLR4/NF - κB signaling pathway, thereby influencing the inflammatory response within the tumor microenvironment and facilitating the tumorigenic progression.

Multiple studies have investigated the involvement of the miR-361-3p/HMGA1 axis in NSCLC progression and its connection to N6-methyladenosine (m6A)-modified lncRNA-NEAT1 (Qi et al., 2023). Firstly, based on the finding that lncRNA-NEAT1 is upregulated in NSCLC, it was found that lncRNA-NEAT1 is associated with low survival rates in NSCLC patients. Human methyltransferase 3 (METTL3)-mediated m6A modification stabilizes and upregulates lncRNA-NEAT1 expression. Secondly, functional experiments showed that METTL3 (Lin et al., 2016) and lncRNA-NEAT1 depletion induce cell apoptosis (Peng et al., 2017), inhibit cell proliferation, and epithelial-mesenchymal transition (EMT) (Wang W. et al., 2023). At the same time, Fabiana Lüönd’s team found in their research (Lüönd et al., 2021) that EMT has the ability to increase the ability of tumor cells to resist immune clearance.

The promotion of cancer cell EMT by lncRNA-NEAT1 suggests its involvement in tumor immune evasion. Research has uncovered the molecular mechanism behind this process, demonstrating that lncRNA-NEAT1 facilitates NSCLC tumorigenesis by acting as a miR-361-3p sponge to increase the expression of high mobility group AT-hook 1 (HMGA1) (Jun⁃feng, 2023). Other studies have shown that patients with severe lung infections have higher expression levels of serum NEAT1 and miR-31, and the differences are statistically significant. Gram-negative bacteria are the primary culprits for pulmonary infections in individuals diagnosed with lung cancer. Notably, there is a notable increase in the levels of serum lncRNA-NEAT1 and miR-31 in these patients, indicating elevated expression. The levels of these molecules are associated with tumor staging, progression, and infection severity (Ma et al., 2020). Therefore, downregulating the expression of lncRNA-NEAT1 can alleviate the symptoms of NSCLC and ultimately achieve therapeutic effects.

In the tumor microenvironment, lncRNAs not only participate in CTL exhaustion but also affect CTL proliferation by participating in other signaling pathways. CTLs have cytotoxic effects on NSCLC tumor cells. For example, in NSCLC, The expression of lncRNA-NEAT1 is markedly upregulated and exhibits a strong correlation with both clinical stage and the presence of lymph node metastasis. LncRNA-NEAT1 can also bind to DNMT1 to inhibit the cGAS/STING signaling pathway. Suppression of lncRNA-NEAT1 in NSCLC cells leads to the activation of the cGAS/STING signaling pathway, resulting in enhanced expression of CXCL10, CCL5, and IFNβ. IFNβ, as type I IFN, activates the type I IFN signaling pathway and promotes CTL proliferation to kill NSCLC tumor cells and achieve good efficacy. Sun Chengcao and Li Shujun et al. (Sun et al., 2016) Studied whether lncRNA-NEAT1 can promote NSCLC progression by regulating the miR-377-3p-E2F3 pathway. RNA pull-down combined with luciferase reporter gene assays, along with bioinformatics investigations, were employed to conduct research in this study. In NSCLC cells, the ceRNA activity of lncRNA-NEAT1 was demonstrated, which opposes the function of has-miR-377-3p. This ultimately leads to the derepression of its endogenous target E2F3. The study also found that BLIMP1 has a significant impact on the miR-377-3p-E2F3 pathway, and discovered that the correlation between lncRNA-NEAT1 and BLIMP1 (B Lymphocyte-Induced Maturation Protein 1) affects the function of immune cells, thereby influencing the immune escape and progression of NSCLC tumor cells, and further participating in the regulation of the tumor immune environment. These findings are of great significance for understanding the tumor immune microenvironment, and provide a new perspective for understanding the molecular mechanisms of lung cancer progression.

#### 4.6.4 Hepatocellular carcinoma

HCC is the most common liver cancer (Mejia and Pasko, 2020). Primary liver cancer, known as HCC, holds the title of being the most prevalent liver cancer and ranks as the third highest contributor to cancer-related fatalities globally. HCC exhibits high malignancy and has a propensity for metastasis and recurrence.

In the HCC database screening analysis, it was found that lncRNA-NEAT1 may target miR-204 to restore the expression level of autophagy-related genes, thereby promoting HCC cell autophagy (Rui et al., 2023). The study showed that lncRNA-NEAT1 can directly bind miR-124-3p, promoting high expression of adipose triglyceride lipase, free fatty acid and diacylglycerol, and activating PPARα to promote the occurrence and development of HCC (Liu et al., 2018), therefore, lncRNA-NEAT1 may become a new therapeutic target for HCC.

The tumor-associated lncRNAs that may has a significant impact on replicative and stress-induced senescence were identified using the GSE77675, GSE116761, and GSE144510 datasets (Chen et al., 2023). In the GSE77675 and GSE116761 datasets, young and old human fibroblasts were examined, while H2O2 and doxorubicin (DOXO) (Lee et al., 2022) were employed to induce premature senescence in human fibroblasts and human CRC cell lines (HCT116 cells) for the GSE116761 and GSE144510 datasets. By analyzing these datasets, a subset of downregulated lncRNAs in senescent cells was revealed through a Venn diagram, combining the data from GSE77675, GSE116761, and GSE144510 (Chen et al., 2023). Notably, comprehensive analysis indicated that NEAT1 was the only lncRNA significantly downregulated during both replicative and stress-induced cellular senescence. Consequently, targeting the expression of lncRNA-NEAT1 has the potential to hinder the growth, mobility, and invasion of hepatocellular carcinoma cells.

The research conducted by Ruru from Zhengzhou University (Chen et al., 2019) focused on investigating the role and mechanism of lncRNA-NEAT1-SFPQ in HCC, the expression levels of SFPQ and IL-8 were analyzed at the transcriptional and translational levels after knocking out or knocking in lncRNA-NEAT1 using Western blot and real-time quantitative PCR experiments. The interactions between lncRNA-NEAT1 and SFPQ, as well as SFPQ and the IL-8 promoter, were analyzed using ChIP and RNA pull-down experiments. Macrophage chemotaxis experiments were performed to evaluate the impact of lncRNA-NEAT1 knockout or overexpression on the migratory ability of HCC cells towards macrophages. The findings demonstrated that knocking out lncRNA-NEAT1 significantly reduced the chemotactic migration of QGY-7703-induced RAW264.7 macrophages, while knocking in lncRNA-NEAT1 significantly increased the chemotactic migration of HuH-7 cells-induced macrophages. Therefore, it was concluded that lncRNA-NEAT1 can regulate HCC tumor cell immune modulation by regulating IL-8 transcription levels and immune cells. Thus, by exerting an influence on the tumor microenvironment of HCC cells, lncRNA-NEAT1 actively contributes to immune modulation in HCC.

In the study by Lian Gang Liao Kai’s team (Steel making et al., 2017) on the effects of lncRNA-NEAT1 on HCC cell apoptosis, in order to evaluate the expression of lncRNA-NEAT1 HCC tumor tissues, real-time quantitative polymerase chain reaction (QRT-PCR) was employed before and after undergoing transcatheter arterial chemoembolization (TACE) treatment. The HepG2 (human liver cancer cells) viability was reduced, caspase-3 (CASP3) activity was increased, compared to the group treated with an irrelevant sequence, the interference of lncRNA-NEAT1 using siRNA led to upregulation of Bax protein expression and downregulation of Bcl-2 expression. Therefore, it was concluded that lncRNA-NEAT1 can inhibit HCC cell apoptosis and may serve as a biomarker for HCC development and a new target for prevention and treatment. Therefore, in clinical practice, we can make a preliminary judgment of the development level of HCC by observing the expression level of lncRNA-NEAT1. In a study (Chen et al., 2023) of lncRNA-NEAT1 and HCC cell senescence, lncRNA-NEAT1 is highly expressed in tumor tissues and hepatocellular carcinoma cells, while the expression of CDKN2A, a biomarker of aging encoding p16INK4a and p14ARF proteins, is decreased, demonstrating a negative correlation. In senescent HCC cells, lncRNA-NEAT1 is reduced, while lncRNA-NEAT1 deficiency leads to senescence in cultured HCC cells. During senescence, lncRNA-NEAT1 is able to translocate into the cytoplasm and interact with the motor protein KIF11, leading to the degradation of KIF11 protein, thereby increasing the expression of CDKN2A in HCC cells. It is further confirmed that lncRNA-NEAT1 inhibits cellular senescence in HCC through KIF11-dependent CDKN2A inhibition, and targeted regulation of lncRNA-NEAT1or KIF11-induced hepatocyte senescence is a potential therapy to inhibit the development of HCC. In the study (Pan J. et al., 2022) of Pan et al., it has been found that overexpression of lncRNA-NEAT1 promotes the migration and invasion ability of HCC cells, and also promotes the transcriptional activity of PKM2, and the knockdown of lncRNA-NEAT1 can inhibit the transcriptional activity of PKM2. When FOXP3 is knocked out, the expression of PKM2 is also weakened, which confirms the regulatory relationship between FOXP3 and PKM2. It is further confirmed that the expression of lncRNA-NEAT1 and PKM2 is positively correlated, and there is colocalization between lncRNA-NEAT1 and FOXP3, and lncRNA-NEAT1 promotes the proliferation and metastasis of hepatocellular carcinoma by regulating the FOXP3/PKM2 axis. Mou et al. (2020) show that Bcl-2-related transcription factor 1 (BCLAF1) directly interacts with the lncNEAT1 promoter and increases lncRNA-NEAT1 expression, and BCLAF1 promotes the proliferation and invasion of HCC cells by targeting lncRNA-NEAT1. Unraveling the relationship between BCLAF1 and lncRNA-NEAT1 and finding the deep mechanism of the interaction between the two will help to understand its function more comprehensively, and ultimately find new ways to treat human cancer. Chronic hepatitis C virus (HCV) infection is a leading cause of HCC.In one study (Tripathi et al., 2022), it has been found that lncRNA-NEAT1 levels increase after HCV infection. MiR-9-5p is one of the lncRNA-NEAT1 targeted targets, which modulates BGH3 mRNA levels. The study also confirms that when miR-9-5p levels are downregulated and BGH3 levels are upregulated after HCV infection, lncRNA-NEAT1 knockout can increase miR-9-5p levels and decrease BGH3 levels, thus indicating LncRNA NEAT1 Regulation of HCV-induced HCC by modulating the miR-9-BGH3 axis.

## 5 New drug development targeting lncRNA-NEAT1

Understanding the mechanism of lncRNA-NEAT1 in tumor immune regulation can provide theoretical support for innovative treatment methods. In addition, in recent years, the mode of operation of lncRNA-NEAT1 in varying immune diseases has been continuously discovered. Therefore, the development of targeted drugs based on lncRNA-NEAT1 is of great significance for the treatment of immune diseases.

At present, there is no relevant experiment to accurately identify the therapeutic agent lncRNA-NEAT1 inhibitor targeting lncRNA-NEAT1, but the related mechanism of lncRNA-NEAT1 In the realm of immune system malfunctions has inspired us to develop new ideas for drug development and disease treatment.

The results showed that targeting non-overlapping sites by leading ASO and the second ASO could effectively inhibit tumor growth in orthotopic HCC xenograft mouse models. In addition, ASO-based PKM splicing conversion therapy also showed inhibitory effects on HCC growth (Ma et al., 2022). In addition, studies have shown that lncRNA-NEAT1 can directly bind to miR-124-3 p, thereby promoting the occurrence and development of HCC *in situ* (Zhu et al., 2021). Therefore, by designing drugs to interfere with the binding of miR-124-3p to lncRNA-NEAT1, it can hinder its manifestation in HCC and further inhibit the development of HCC. At the same time, the experimental also proved the expression level of lncRNA-NEAT1 was significantly increased in BC. Silencing its expression may suppress the growth and spread of breast cancer cells by inhibiting the polarization of M2 tumor-associated macrophages (TAMs) (Zou and Yang, 2020). Therefore, we can treat BC by inhibiting the expression of lncRNA-NEAT1 to make BC tumor cells more easily recognized and cleared by immune checkpoint inhibitors.

Reducing the expression of lncRNA-NEAT1 may combine drugs targeting lncRNA-NEAT1 with traditional chemotherapeutic drugs. The presence of lncRNA-NEAT1 affects the expression level of immune checkpoint molecules molecules (Example PD-L1) on tumor cells. When we treat them by targeting lncRNA-NEAT1, the expression levels of these molecules can be reduced, thereby weakening the ability of tumor cells to evade immune system attacks. The combination of drugs targeting lncRNA-NEAT1 and immunotherapy (such as PD-1/PD-L1 inhibitors) can produce synergistic anti-tumor effects. For example, the combination of anti-PD1 antibody or its antigen binding fragment with drugs such as taxanes and platinum compounds can reduce the expression level of lncRNA-NEAT1, thereby increasing the sensitivity of tumor cells to immune checkpoint inhibitors and making it easier to be acknowledged and cleared by the immune system. This combination therapy strategy can be used to treat diseases such as esophageal squamous cell carcinoma or esophageal adenocarcinoma, and improve the efficacy through the synergistic effect of two different drugs, may improve the sensitivity and effect of treatment. Combination of drugs for different targets (such as drugs for lncRNA-NEAT1 combined with PI3K/AKT/mTOR pathway inhibitors) to increase anti-tumor effects. This multifaceted combination of treatments provides a more effective strategy for cancer treatment.

In terms of inflammation, since lncRNA-NEAT1 reduces the functionality of miRNAs (such as miR-9-5p, miR-125a) by acting as miRNA sponges, giving these miRNAs mimics or stabilizers may help restore their regulation of target genes. Studies have displayed that reducing the expression of lncRNA-NEAT1 can reduce pro-inflammatory factors induced by TNFα-induced protein (TiP) and reverse osteolysis induced by overexpression of Bruton tyrosine kinase (BTK). In response to this finding, scientists have developed si-NEAT1 PLGA-based microparticles for local injection to treat mouse skull dissolution models. Another method is to develop a nanoparticle delivery system to package drugs that inhibit lncRNA-NEAT1 or miRNA modulators. This system can ensure that the drug persists in the body and performs better than natural or synthetic drugs. Nanoparticle systems can accurately deliver drugs to infected and inflammatory sites, thereby improving treatment efficiency and reducing systemic side effects. Through this strategy, we can achieve more accurate drug treatment, improve the therapeutic effect, and reduce the adverse reactions of patients. In addition, drugs targeting the lncRNA-NEAT1-related TLR2/NF-κB signaling pathway can also be developed to reduce LPS-induced inflammatory responses. These drugs can target specific signaling pathways, inhibit the inflammatory process from the source, and provide new treatment options for the treatment of inflammation-related diseases (Han, 2013). For example, NF-κB signaling pathway inhibitors may help to slow down the inflammatory response mediated by lncRNA-NEAT1 (Lin et al., 2022). The development of drug inhibitors for these pathways helps to control NEAT1-mediated inflammation. These treatments have the potential to improve targeting and reduce systemic side effects of inflammatory treatments.

In autoimmune diseases, the expression of lncRNA-NEAT1 in peripheral blood is abnormally increased in patients with SLE. Therefore, regulators of the lncRNA-NEAT1/B cell activating factor/IFN pathway may help alleviate SLE symptoms (Zhang and Huang, 2022). We can design potential drug targets to target the regulation of lncRNA-NEAT1 in different diseases. In SLE, lncRNA-NEAT1 targets the JAK-STAT signaling pathway by binding to miR-365a-3p, thereby inhibiting its promotion of IL-6 secretion, which helps to improve SLE symptoms. In OA, lncRNA-NEAT1 regulates the apoptotic process of chondrocytes, so drugs that enhance the activity of lncRNA-NEAT1 may help protect articular cartilage. Studies have shown that demolition of lncRNA-NEAT1 can noticeably shorten the survival time of human tumor tissue xenograft (PDX) model mice. In MS, deletion of B cells can inhibit T cell proliferation and reduce the secretion of pro-inflammatory cytokines, while also reducing the number of TH17 cells. At present, some treatment methods for CD20 + B cells have been developed (Zhang C. et al., 2021) and deletion therapy has been applied to MS. Therefore, the study of knockdown therapy targeting lncRNA-NEAT1 may provide a choice for the treatment of MS. Considering the characteristics of the disease, for specific skin inflammation such as psoriasis, local administration strategies such as ointments, gels (Fan et al., 2022), patches or nano-technology drug delivery systems will be more suitable for disease-related expression issues.

As a disease predictive marker, lncRNA-NEAT1 offers a fresh perspective on immunotherapy and has a crucial impact on cancer, Immune response and inflammatory reaction diseases. Inhibition of its expression by targeted drug design can treat a variety of diseases. According to the characteristics of different diseases, different administration strategies can improve the therapeutic effect and reduce the side effects. It has potential clinical application value in different diseases, and is expected to become the focus of exploring tumor immune mechanism and drug research and development, which has important clinical significance.

## 6 Discussion and summary

In summary, this study has thoroughly investigated the role of lncRNA-NEAT1 in the immune system and associated diseases. LncRNA-NEAT1 is not only involved in the regulation of immune cell differentiation and function but also plays a critical role in the development of various diseases, including autoimmune disorders, inflammatory responses, and tumors. LncRNA-NEAT1 regulates both innate and adaptive immunity through multiple mechanisms. In innate immunity, it modulates the activation of signaling pathways such as RIG-I, which is essential for the body’s first line of defense against pathogen invasion. In adaptive immunity, it influences T cell function, particularly in apoptosis and the balance between Th17 and Treg cells, thereby affecting inflammatory and autoimmune responses. The diverse expression patterns of lncRNA-NEAT1 across different diseases present significant potential for diagnostic and therapeutic applications. However, further research is necessary to fully elucidate its precise mechanisms in various cell types and disease states. Future studies should aim to translate foundational research findings into clinical applications through preclinical and clinical trials to assess the efficacy and safety of potential therapies targeting lncRNA-NEAT1.
